# Intranodal palisaded myofibroblastoma: a case report

**DOI:** 10.1186/1757-1626-3-45

**Published:** 2010-02-02

**Authors:** Helen Karvouni, Anneza I Yiallourou, Maria Kyriazi, Vaia Stafyla, Vassilis Smyrniotis, Agathi Kondi-Pafiti

**Affiliations:** 1 Department of Pathology, Aretaieion Hospital, University of Athens, Vasilissis Sophias 76, 11528, Athens, Greece; 2 2nd Department of Surgery, Aretaieion Hospital, University of Athens, Vasilissis Sophias 76, 11528, Athens, Greece

## Abstract

Intranodal palisaded myofibroblastoma is a rare benign soft tissue tumor, almost always arising from inguinal lymph nodes. It usually presents as a painless, slow-growing inguinal mass. We report herein a case of an intranodal palisaded myofibroblastoma occurring in a 36-year-old man. The salient clinicopathologic features of this unusual tumor are presented and the literature is briefly reviewed.

## Introduction

Intranodal palisaded myofibroblastoma (IPM) also called as intranodal hemorrhagic spindle cell tumor with amianthoid fiber is a rare benign tumour of the lymph node that may be derived from myofibroblasts or smooth muscle cells. The most usual area of presentation is the inguinal lymph nodes, but occurrence within other areas such as mediastinum and submandibular lymph nodes has also been reported [1]. This unusual lesion was first well characterized in 1989. Previous reports of similar cases in the literature were considered to represent intranodal primary or metastatic neurilemoma. To our knowledge, approximately 53 such cases have been reported in the literature [2]. The ages of patients ranged from 19 to 78 years with a slight male predominance [3,4].

## Case presentation

**Figure 1:**
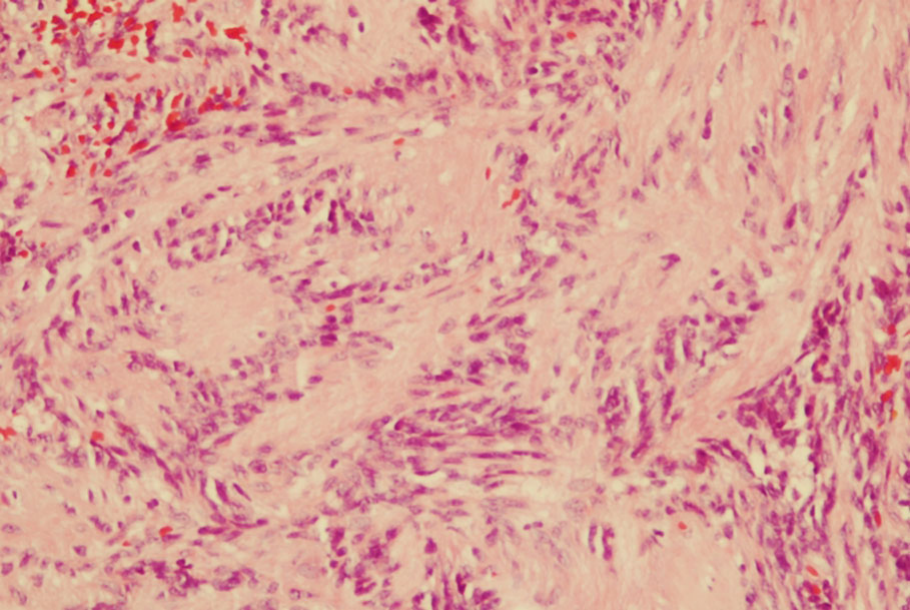
**Intranodal palisaded myofibroblastoma with a thin rim of residual lymph node at the periphery (hematoxylin-eosin ×25)**.

**Figure 2:**
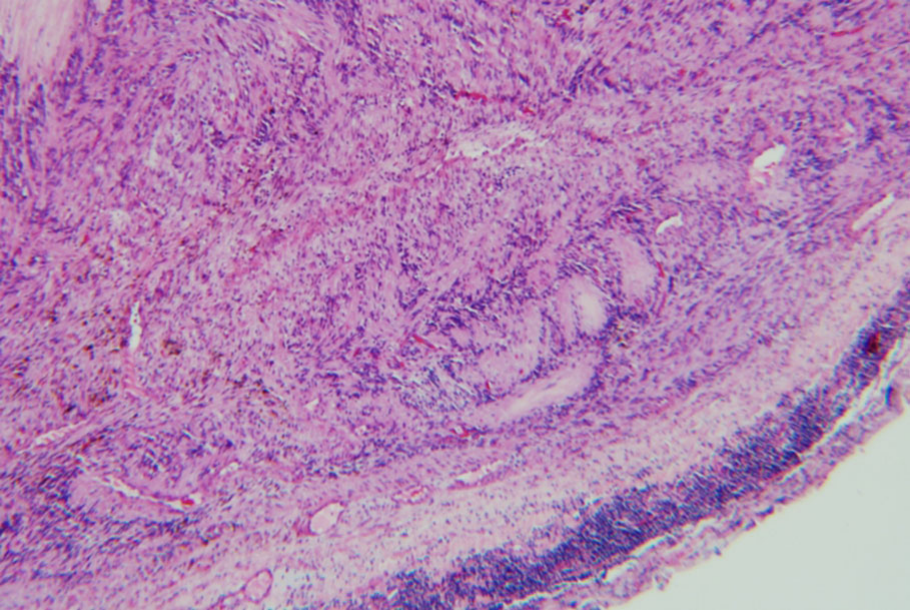
**Amianthoid fibers (hematoxylin-eosin ×100)**.

**Figure 3:**
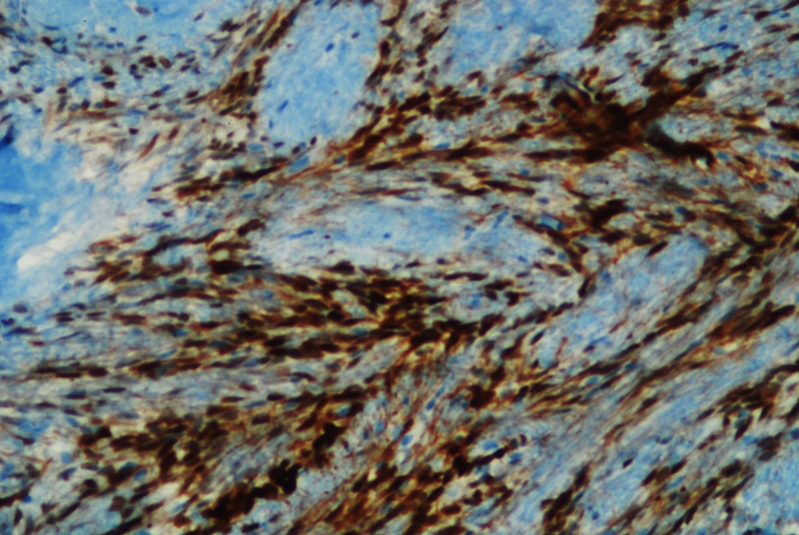
**Cyclin D1 positive tumor cells (immunohistochemistry ×100)**.

A 36-year-old Greek Caucasian male patient presented with a slowly growing lump in his right groin. He mentioned that he first noticed the swelling two years ago. There were no symptoms associated with it. In physical examination, a solitary, painless, mobile, firm mass was determined in the right inguinal region of the patient. He had no other significant findings in physical examination or clinical work-up. The lesion was excised for diagnostic purposes. The specimen consisted of a well circumscribed mass measuring 2.5 cm in diameter. Its cut surface was whitish, nodular and firm. Representative sections of the specimen revealed a lymph node almost replaced by a spindle cell tumor (Figure 1) consisting of cells with bland nuclear features and no significant mitotic activity. In several areas, nuclear palisading was noted and in these areas the tumor was reminiscent of a schwannoma. Focal stellate-shaped acellular eosinophilic structures were identified in the stroma (Figure 2). Immunohistochemical analysis showed that the neoplastic cells were positive for cyclin D1 (Figure 3) and smooth muscle actin, whereas they were negative for S-100 protein, cytokeratin, melan A, CD34, desmin and EMA. In this clinical setting, the morphology of the tumor along with its immunohistochemical findings were characteristic for an intranodal palisaded myofibroblastoma.

## Discussion

Intranodal palisaded myofibroblastoma is a benign tumor which has a great predilection for the inguinal lymph nodes. Submandibular and cervical lymph nodes have also been reported as rare originating sites [3]. It was first well characterized in 1989 in two series of patients that were reported in the literature under the names of palisaded myofibroblastoma [5] and intranodal hemorrhagic spindle cell tumor with amianthoid fibers [6]. Clinically, it presents as an asymptomatic, slowly growing tumor. It is more common in males and it is diagnosed at a wide age range (from 19 to 78 years) [3]. Its size varies from 0.6 to 5 cm [4].

Its predominant morphologic features include the bland appearance of its constituent spindle cell population and the presence of acellular eosinophilic stellate areas (so-called amianthoid fibers). As an intranodal lesion, intranodal palisaded myofibroblastoma grows from the interior portion of the lymph node expanding outwardly to the periphery; thus, normal lymphoid tissue is compressed to the periphery.

The immunohistochemical profile of the neoplastic cells along with their ultrastructural features is indicative of myofibroblastic or smooth muscle differentiation [5,6]. Intranodal palisaded myofibroblastoma has recently been shown to have a strong expression for cyclin D1 and a low proliferating index of Ki- 67 [7]. The overexpression of cyclin D1 presented in the study of Kleist et al, points to the proliferation regulatory pathway as one of the factors involved in the etiologic pathogenesis of intranodal palisaded myofibroblastoma [7]. It is negative for neural markers such as S100, and endothelial markers such as CD34, and desmin.

The presence of a spindle cell neoplasm in a lymph node requires a thorough clinical work - up to identify a primary neoplasm. Metastatic spindle squamous cell carcinoma, melanoma, sarcomatoid renal cell carcinoma are examples of carcinomas that can present as spindle cell lymph node lesions. Metastatic sarcomas should also be included in the differential diagnosis. Spindle cell tumors that arise primarily in lymph nodes are Kaposi sarcoma, dendritic cell tumor and benign mesenchymal tumors such as neurofibroma [5]. Schwannomas are unlikely to occur within the lymph nodes, due to the fact that they arise from Schwann cells of the nerve sheath, which do not innervate lymph nodes. Furthermore, intranodal palisaded myofibroblastoma lacks Antoni A type A and B areas and does not express S100 protein. Nuclear palisading, amianthoid-like changes and the lack of a history of immunocompromised status distinguish intanodal palisaded myofibroblastoma from Kaposi sarcoma. In order to exlude hemangioendothelioma from the differential diagnosis, expression of endothelial markers such as CD34 and CD31 should be performed during the immunohistochemical analysis. Finally, one must rule out the possibility of metastases from malignant melanoma and sarcoma based on thorough clinical history and an appropriate immunohistochemical profile.

Although a wide range of soft tissue tumors can be included in the differential diagnosis, intranodal palisaded myofibroblastoma has a distinctive morphological feature and an immunohistochemical profile. When the diagnosis is suspected, it is best to report this lesion as a low grade spindle cell tumor and recommend surgical excision. Excellent prognosis has been reported after surgical treatment with a 6% recurrence rate and no malignant transformation [3].

## Consent

Written informed consent was obtained from the patient for publication of this case report and accompanying images. A copy of the written consent is available for review by the journal's Editor-in-Chief.

## Competing interests

The authors declare that they have no competing interests.

## Authors' contributions

HK and AKP performed histopathological and immunohistochemical analysis and contributed substantially to pathology content. AY drafted the manuscript. MK and VS were responsible for critical revision of scientific content and helped in drafting the manuscript. VS was the surgeon and approved the final version of the manuscript for publication. All authors have read and approved the final version of the manuscript.
